# Combined assessment of EGFR pathway-related molecular markers and prognosis of NSCLC patients

**DOI:** 10.1038/sj.bjc.6604781

**Published:** 2008-12-02

**Authors:** M I Galleges Ruiz, K Floor, S M Steinberg, K Grünberg, F B J M Thunnissen, J A M Belien, G A Meijer, G J Peters, E F Smit, J A Rodriguez, G Giaccone

**Affiliations:** 1 Department of Medical Oncology, VU University Medical Center, Amsterdam, The Netherlands; 2 Biostatistics and Data Management Section, National Cancer Institute, Bethesda, MD, USA; 3 Department of Pathology, VU University Medical Center, Amsterdam, The Netherlands; 4 Department of Pulmonary Diseases, VU University Medical Center, Amsterdam, The Netherlands; 5 Medical Oncology Branch, Center for Cancer Research, National Cancer Institute, Bethesda, MD, USA

**Keywords:** EGFR pathway, NSCLC, prognosis, CMET, KRAS, biomarkers

## Abstract

The purpose of this study is to evaluate the prognostic value of the combined assessment of multiple molecular markers related to the epidermal growth factor receptor (EGFR) pathway in resected non-small cell lung cancer (NSCLC) patients. Tumour specimens of 178 NSCLC patients were collected and analysed for EGFR and KRAS mutation status by DNA sequencing, and for EGFR copy number by fluorescent *in situ* hybridisation. Tissue microarrays were generated and used to determine the expression of multiple EGFR pathway-related proteins by immunohistochemistry. We analysed the association between each marker and patient prognosis. Univariate analyses for each clinical variable and each molecular marker were performed using Kaplan–Meier curves and log-rank tests. From these results, we selected the variables KRAS mutations and expression of cytoplasmic EGFR, granular pERK, nuclear pSTAT3, cytoplasmic E-cadherin and cytoplasmic pCMET to enter into a Cox proportional hazards model, along with stage as the strongest clinical variable related with prognosis. Of the EGFR-related markers evaluated here, the markers EGFR, pERK, pSTAT3, E-cadherin, pCMET and mutations in KRAS were associated with survival when analysed in combination in our patient cohort, with *P*=0.00015 as the *P*-value for a test of the additional impact of markers on prognosis, after taking stage into consideration. Confirmation of the impact of these markers in independent studies will be necessary.

Lung cancer is the leading cause of cancer-related deaths worldwide (Jemal *et al*, 2006), and non-small cell lung cancer (NSCLC) represents 85% of lung tumours. The epidermal growth factor receptor (EGFR) pathway plays a fundamental role in the carcinogenesis and progression of various tumour types, including NSCLC (Hynes and Lane, 2005). Epidermal growth factor receptor (ErbB-1) is a member of the ErbB family of receptors, which also includes HER2/-neu (ErbB-2), Her 3 (ErbB-3) and Her 4 (ErbB-4). Autophosphorylation of EGFR intracellular tyrosine kinase domain results in activation of several downstream signalling pathways, including the PI3K, STAT and the mitogen-activated protein kinase pathways pathways, which regulate biological responses such as proliferation, cell motility, angiogenesis, cell survival and differentiation (Yarden and Sliwkowski, 2001).

An improved understanding of EGFR signalling has led to the development of anticancer therapeutics directed against EGFR, including the tyrosine kinase inhibitors (TKIs) gefitinib and erlotinib (Giaccone, 2005). Objective responses to these agents are observed in a small subset of unselected NSCLC patients, and several molecules involved in EGFR signalling have been evaluated in an effort to identify markers of TKI sensitivity. Such molecular markers include specific mutations in EGFR or KRAS, *EGFR* gene copy number (Lynch *et al*, 2004; Paez *et al*, 2004; Cappuzzo *et al*, 2005; Giaccone, 2005; Pao *et al*, 2005), the activation status of AKT and STAT signalling pathways (Sordella *et al*, 2004), and the expression level of HER2 (Moasser *et al*, 2001; Hirata *et al*, 2005). More recently, amplification of the CMET receptor (Engelman *et al*, 2007), the expression of epithelial to mesenchymal transition markers, such as E-cadherin and vimentin (Thomson *et al*, 2005; Yauch *et al*, 2005), and the downregulation of HIF-1*α* have also been linked to responsiveness to EGFR-targeted agents (Lu *et al*, 2007).

Although several of these markers have been identified as potential predictors for response to EGFR TKIs in patients with advanced NSCLC, some of them have also been shown to be prognostic for survival, irrespective of treatment. It is important to be able to distinguish between these two effects.

The presence of EGFR mutations has been proposed to be a positive prognostic factor (Eberhard *et al*, 2005), whereas high-EGFR copy number and the presence of KRAS mutations have both been associated to poor prognosis in resected NSCLC patients (Nelson *et al*, 1999; Hirsch *et al*, 2003; Massarelli *et al*, 2007). Several other markers have also been associated with poor prognosis in NSCLC (EGFR, CMET, E-cadherin, pAKT (Takanami *et al*, 1996; Bremnes *et al*, 2002; David *et al*, 2004; Deeb *et al*, 2004; Masuya *et al*, 2004a)), but at present there is no single marker that can be used to guide therapy or predict prognosis of NSCLC patients.

We hypothesised that the combined analysis of several of these molecular markers (Figure 1), which provides information on the activity/sensitivity of the EGFR signalling pathway at different points, is related to the prognosis of NSCLC patients when analysed in combination. Such analysis aids to distinguish the prognostic implication of the EGFR pathway from its predictive value in patients treated with agents targeted to this pathway. Thus, we carried out an analysis of EGFR and KRAS mutational status, EGFR copy number and the expression of EGFR, HER2, pCMET, pAKT, PTEN, pSTAT3, pSTAT5, pERK, HIF-1*α*, E-cadherin and vimentin, in resected NSCLC patients to assess their potential combined prognostic significance with respect to overall survival.

## Materials and methods

### Patients and samples

Radically resected tumour specimens of 178 NSCLC patients were collected. For 148 patients, both frozen and paraffin-embedded tissue was available; for 30 patients only paraffin-embedded material was available. Samples were obtained from patients with pathological stage I, II or III and 24% of patients received (neo-)adjuvant chemo/radiotherapy. A full description of patient characteristics is provided in Table 1. The study was carried out in accordance with the medical ethical committee guidelines of VU University Medical Center, Amsterdam, The Netherlands.

### Isolation of genomic DNA

DNA was isolated from frozen tissue (*n*=148). Sections of tissue samples flanking those used for DNA isolation were verified by the study pathologists (KG and FBJMT) to contain at least 50% of tumour cells. Genomic DNA was extracted from frozen samples using trizol, following manufacturer instructions (Life Technologies, Breda, The Netherlands).

### PCR amplification and DNA sequencing

Mutation analysis was carried out on 148 patients for which frozen tissue samples were available, because paraffin-embedded samples might yield a higher proportion of false-positive results (Marchetti *et al*, 2006; Gallegos Ruiz *et al*, 2007a), and we avoided using two different sources of samples (frozen and paraffin) for one type of analysis. We used 100 ng of genomic DNA derived from tumour cells as template in nested PCR reactions to amplify DNA fragments corresponding to exons 18–21 of EGFR, and exons 1 and 2 of KRAS. The PCR protocol and the sets of primers have been described in detail earlier (Janmaat *et al*, 2006). Polymerase chain reaction products were purified using a presequencing kit (Amersham Biosciences, Roosendaal, The Netherlands), and sequenced with both forward and reverse primers using the BigDyeTM Terminator v3.1 Cycle Sequencing Kit (Applied Biosystems, Foster City, CA, USA), using the ABI PRISM™ 3100 Genetic analyzer (Applied Biosystems). Non-amino acid changing mutations were defined as single nucleotide polymorphisms. Mutations were confirmed by sequencing independent PCR products.

### Fluorescent *in situ* hybridisation

Fluorescent *in situ* hybridisation (FISH) was performed only on frozen sections, as this analysis provides poorer results on paraffin-embedded tissue (Gallegos Ruiz *et al*, 2007b). Frozen sections of 4 *μ*m thickness were fixed with methanol/acetic acid (3 : 1) and pretreated by digestion with 0.01% pepsin/0.2N HCl at 37 °C for 2 min, and incubated for 10 min in 50 mM MgCl2/PBS followed by 10 min in 50 mM MgCl_2_/3.7% formaldehyde/PBS. After 2 h incubation with 70% formamide/0.6 × SSC, the sections were dehydrated with alcohol. Following pretreatment, 10–15 *μ*l LSI EGFR spectrum orange/CEP7 Spectrum Green probe (Vysis, Abbot Laboratories, Downers Grove, IL, USA) was applied, the section was covered with a coverslip and sealed with rubber cement. Following a denaturation step at 80 °C for 10 min, slides were placed in a humidified chamber at 37 °C for 20–24 h. Then, sections were washed with 1.5 M urea/0.1 × SSC at 45 °C for 30 min, and with 2 × SSC for 2 min. Finally, sections were counterstained with 4′,6-diamidino-2-phenylindole (DAPI, Sanbio BV, Uden, The Netherlands), dehydrated with alcohol, air-dried and mounted using Vectashield (Brunschwig Chemie, Amsterdam, The Netherlands).

### Scoring of FISH analysis results

FISH slides were evaluated using a Leica DMRA fluorescent microscope (Leica Microsystems BV, Wetzlar, Germany) with a × 60 PL Fluotar oil immersion objective (NA=1.40). Scoring was done by two independent observers (KF and MIGR). For every sample, the complete section was screened for homo/heterogeneity of the FISH signals. The signals in 200 tumour cells were counted in at least three representative microscopic fields. The number of cells having 0, 1, 2, 3, 4, 5 or ⩾6 red signals or clusters was noted, and samples were categorised as described earlier (Hirsch *et al*, 2005). Samples were considered as having high-EGFR polysomy when ⩾4 dots per nucleus were present in ⩾40% of tumour cells, and as having EGFR amplification when tight EGFR gene clusters were present in ⩾10% of cells.

### Tissue microarray construction

Paraffin-embedded tumour material of 178 patients was cut into 4 *μ*m-thick sections and placed onto glass slides. Slides were stained with hematoxylin and eosin, and a pathologist (KG) verified the presence of tumour cells and marked the tumour area. Biopsies of 0.6 mm diameter were taken from the donor block, two from the tumour and one from the normal tissue area surrounding the tumour. Biopsies from the donor blocks were included in recipient tissue array blocks using a precision tissue array instrument (Beecher Instruments, Sun Prairie, WI, USA).

### Immunohistochemistry

Tissue microarray (TMA) sections were deparaffinised using xylene and dehydrated in alcohol. To block the endogenous peroxidase activity, tissue slides were incubated in methanol/0.3% H_2_O_2_ for 30 min. Antigen retrieval was carried out by heating the slides in 0.1 M sodium citrate or 1 mM Tris/ethylene diamine tetraacetic acid (pH 9.0) for 30 min. Sections were then incubated with the primary antibody overnight at 4 °C, using sections incubated with antibody diluent (Immunologic, Duiven, The Netherlands) as negative control. Sections were developed using the DAKO Envision™ visualisation system (Dakocytomation, Heverlee, Belgium). Pretreatment conditions and antibody dilutions are available on request. The phosphorylation status of CMET and AKT at several residues was analysed using different phospho-specific antibodies.

### Immunohistochemistry scoring

Protein expression determined by immunohistochemistry (IHC) was evaluated using an Olympus BX50F bright field microscope (Olympus Optical Co Ltd., Tokyo, Japan) with a × 40 plan objective (NA=0.65). Scoring was done by two observers (KF and MIGR). For each protein, intensity (negative: 0, weak positive: 1, moderately positive: 2, strong positive: 3) and percentage of positively stained cells were scored. Some cases suffered tissue loss or lack of tumour cell representation to an extent that precluded the evaluation of protein expression. The different subcellular localisation of the proteins was recorded as nuclear (N), membrane-associated (M) or cytoplasmic (C) (Cheuk and Chan, 2004). For example, a marker described as pCMET.1003.N refers to CMET phosphorylated at residue 1003 localised in the nucleus. We also note that, in the particular case of pERK, some samples showed specific pERK granules in the cytoplasm and were categorised as pERK.gr. The staining intensity value was multiplied by the percentage of positive cells (Lagendijk *et al*, 1998), yielding a final expression score ranging from 0 to 300.

### Statistics

Univariate analyses were done, using standard two-tailed log-rank tests and individual Kaplan–Meier curves, to serve as a screening procedure to initially determine which parameters should be considered for evaluation in a Cox proportional hazards model (Kaplan and Meier, 1958; Mantel, 1966; Cox, 1972). Groupings for analyses were formed on the basis of the observed distribution of the values. After these initial groupings had been done, all of the clinical, demographic and marker parameters were initially evaluated in a univariate fashion with respect to survival. In a limited number of cases, when the results of an exploratory evaluation identified that there could be a difference in prognosis by further combining the categories into just two groups of patients, based on one of the two or three possible cut-points, the resulting *P*-value was adjusted by multiplying the unadjusted *P*-value by the number of implicit tests performed in order to arrive at the final division. For example, if the initial Kaplan–Meier analysis used data in three groups, and two were similar in prognosis, but one differed from the other two, then the data were regrouped into the two resulting categories, and the adjusted *P*-value was reported to be two times the unadjusted one, as there were two possible groupings evaluated implicitly, with the best one selected for further evaluation.

The significance for survival of the different parameters was analysed in a multivariate analysis using Cox proportional hazards modelling. Only those parameters with unadjusted *P*-values of <0.15 from the univariate analyses were included in an initial Cox model. A standard backward selection technique, as well as stepwise selection, was used to identify parameters to be considered of joint importance in the model. In addition, a likelihood ratio test was performed to determine the increase in a given Cox model's predictive ability following inclusion of marker values. All *P*-values are two-tailed, and except as noted above, have not been adjusted for multiple comparisons. As this is an exploratory study and not definitive, the findings are intended to suggest the strength of the evidence that a marker may be associated with prognosis, and to indicate which markers may be given greater priority for confirmation in later studies.

## Results

### EGFR, KRAS mutations and EGFR copy number

EGFR mutation analysis was successful in 136 patients and mutations were identified in 4% (*n*=5) of the patients. Mutations observed were P848L (*n*=1), L858R (*n*=1), DelL746-752S (*n*=1), the double mutation E709K+L858R (*n*=1) and the double mutation S768I+L861Q (*n*=1). Epidermal growth factor receptor mutation P848L was not considered to be a cancer-specific EGFR mutation based on previous findings by us and others (deGunst *et al*, 2007; Janne *et al*, 2006). KRAS mutations were observed in 18% (*n*=25) of the 139 patients whose samples could be analysed. Mutations were predominantly observed in codon 12 (*n*=19) but were also detected in codon 13 (*n*=3) and codon 61 (*n*=3). Finally, FISH could be evaluated in 138 patients. Amplification (tight EGFR signal clusters) was observed in 6% of the patients, whereas high polysomy, defined as more than four EGFR signals per cell in more than 40% of cells, was observed in 8% of the patients. Epidermal growth factor receptor copy number and EGFR mutation may be related, as out of five EGFR mutant samples, two samples showed high polysomy and one sample showed EGFR amplification.

### Protein markers

**Figure 2:**
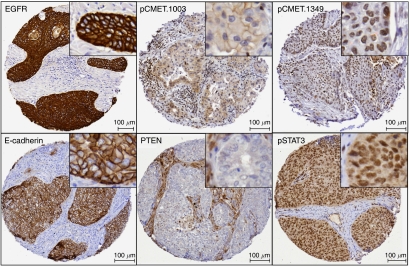
Representative immunohistochemical staining pattern for several of the markers analysed. Examples of positive stainings of total EGFR on the membrane, pCMET.1003 on the membrane, pCMET.1349 in the nucleus, E-cadherin on the membrane, tumour cells negative for PTEN with positive stromal staining and positive pSTAT3 staining in both the cytoplasm and nucleus.

The expression, phosphorylation status and subcellular localisation of proteins evaluated in 178 patients using the TMA by IHC are referred to as protein markers. As described in the Materials and Methods section, for each protein we scored the staining intensity and the percentage of positively stained cells. In Figure 2, representative images of immunohistochemical staining for EGFR (A), pCMET (B-C), E-CADHERIN (D), PTEN (E) and pSTAT3 (F) are shown.

### Univariate analysis

To evaluate the effect of each clinical and molecular or protein markers separately, we performed univariate analyses using Kaplan–Meier curves and log-rank tests. Table 2 presents the results of a univariate evaluation of demographic and clinical parameters, and their association with overall survival. The type of resection (R0 *vs* R1/R2), stage (I *vs* II *vs* III or I *vs* II/III) and use of pretreatment chemo-radiation, each appear to be sufficiently associated with survival to be candidates for consideration as parameters for evaluation in a Cox model. However, because pretreatment chemo-radiation was performed in only three patients, this parameter was excluded from further evaluations.

EGFR FISH and mutations in both EGFR and KRAS were also evaluated to determine whether any of these parameters may be importance in prognosis (Table 2). KRAS was the only one of this class of parameters with any potential association with survival. Surprisingly, the presence of a KRAS mutation was related, although not significantly, with improved survival, which contrasts with previous literature (Van Zandwijk *et al*, 1995; Miyake *et al*, 1999).

**Figure 3:**
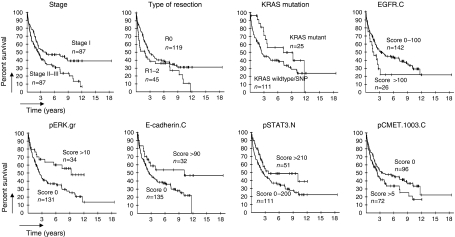
Survival across the strata determined by eight markers. Kaplan–Meier curves showing the effect on survival of univariate analysis of the variables selected to be included in the Cox model.

Individual survival analyses of each protein marker at each staining site were also performed (Table 2). On the basis of cut-off *P*-value of 0.15, the marker parameters, EGFR.C, pERK.gr, pSTAT3.N, E-cadherin.C and pCMET.1003.C are candidates for inclusion in a Cox model. Kaplan–Meier curves of each parameter to be included in the Cox model are shown in Figure 3.

### Multivariable analysis

The univariate analysis showed that both stage (I *vs* II/III) and resection (R0 *vs* R1/R2) were of importance in prognosis. As the two parameters were highly associated with one another (*P*<0.0001 by Fisher's exact test), and by Cox modelling, it was determined that these parameters could not simultaneously be included in the model and have a combined significant impact on survival. When models, which contained either stage or type of resection were constructed, restricted to the set of patients in which type of resection was known, the results of the modeling were quite similar (data not shown). As stage is conventional, widely accepted parameter, the Cox model incorporating stage will be presented to determine if the molecular and protein markers retained their prognostic significance after taking stage into consideration. In Table 3, results from this multivariate analysis are shown using a backward selection. This model is derived from the subset of 116 patients who were not missing any data on the parameters in the model or on the type of resection. Using a stepwise selection model, we were also able to show that pERK.gr was associated with survival adjusting for other markers (data not shown). Further, to show that the markers contributed prognostic information beyond stage, a likelihood ratio test was performed. On the basis of backward selection model, the parameters KRAS mutation, EGFR.C, ECADHERIN.C, pSTAT3.N and pCMET.1003.C added significantly in prognostic ability (*P*=0.00015) beyond stage alone. Thus, these would be markers, which may be of higher priority to consider evaluating in a subsequent, more definitive study.

As the patients included in this study are somewhat heterogeneous with respect to stage, a further subset analysis was performed to determine if there are markers, which are jointly associated with prognosis in patients with stage I disease only, the predominant subset of patients. After a univariate screening procedure was conducted, only pSTAT3.N, EGFR.C and vimentin.N were each associated with a trend towards association with survival (*P*<0.10 for each). When evaluated in a Cox model with backward selection, only vimentin.N emerged as a marginally significant parameter using 0.05 as the traditional threshold for inclusion in a final model (*P*=0.055, hazard ratio=4.10).

## Discussion

At present, the pTNM staging is regarded as the most reliable prognostic factor for NSCLC (Watanabe, 2003). However, staging alone is unable to correctly predict survival in a significant proportion of patients who undergo radical resection. The use of biological markers has been investigated as a way to increase the ability to estimate prognosis in patients. In the study described here, stage was indeed related to prognosis of NSCLC patients, as was type of resection. Immunohistochemical expression of cytoplasmic EGFR, cytoplasmic E-CADHERIN, nuclear pSTAT3, cytoplasmic CMET, granular pERK and KRAS mutations jointly added prognostic significance to stage.

Much effort is currently ongoing to identify biomarkers that are prognostic for survival, and also to identify markers that are predictive of response to systemic therapies. Complementary DNA microarray technology has been widely used to this end, but this technique is still far from clinical implementation mainly due to the need for validation and standardisation across laboratories, and also due to the high costs. Immunohistochemistry, on the other hand, is a widely accepted technique of assessing protein expression with much lower costs than microarrays, although validation and standardisation are also critical issues. However, it is also known that prognostic markers based on IHC can provide inconsistent or contradictory results, owing to the use of different antibodies and processing methods (Atkins *et al*, 2004; Baker *et al*, 2005), as well as different scoring and categorisation systems. All these issues emphasise the need for standardised processing and scoring procedures. In addition, as quantitation is subjective and subcellular localisation may matter (Cheuk and Chan, 2004), it would be desirable to have IHC findings reported carefully and in detail. In this study, we have used resection specimens to perform IHC on TMAs to identify protein expression, and we have scored the staining intensity, the percentage of positive cells and the subcellular localisation of proteins, in order to present our data in the most complete manner.

Given the importance of EGFR-mediated signalling in NSCLC, various EGFR pathway-related proteins have already been studied as potential markers using IHC in NSCLC tumour samples. Several downstream proteins have been shown to provide some prognostic information, such as E-cadherin, EGFR (Bremnes *et al*, 2002; Deeb *et al*, 2004) and pAKT (David *et al*, 2004), although none of them has proven to be sufficiently useful in clinical diagnostics in terms of prediction of response to treatment or prognosis. As simultaneous analysis of various markers could potentially increase prognostic significance over individual markers, we have used a multi-marker approach, using TMAs, to investigate the relevance of several EGFR pathway-related markers and their association with NSCLC patient prognosis.

Here, we also show that high expression of pCMET is related to poor prognosis and high expression of E-cadherin is related to improved outcome (Bremnes *et al*, 2002; Masuya *et al*, 2004b). We found KRAS mutations to be related to favourable prognosis, which contrasts the existing literature that indicates these mutations to be a negative prognostic factor (Van Zandwijk *et al*, 1995; Miyake *et al*, 1999). However, this prognostic effect of KRAS mutations is mainly observed in adenocarcinomas of the lung (Slebos *et al*, 1990; Mitsudomi *et al*, 1991; Sugio *et al*, 1992). Here we analysed the effect on survival of NSCLC patients in a population with both adenocarcinoma (36%) and squamous cell carcinoma (43%) histologies. In this patient cohort, patients with adenocarcinoma histology show improved survival as compared with the squamous cell carcinoma histology. As KRAS mutations were mainly observed in patients with adenocarcinoma histology, this could have been confounding the favourable prognostic effect we observe for KRAS mutations. The negative prognostic effect of KRAS mutations could also not be confirmed in other studies analysing bigger groups of patients with equally balanced adenocarcinoma *vs* squamous cell carcinomas (Moldvay *et al*, 2000; Schiller *et al*, 2001; Tsao *et al*, 2007). We also observed that high expression of nuclear pSTAT3 was related to improved survival. pSTAT3 expression has previously been reported to be associated with smaller tumours and limited smoking history (Haura *et al*, 2005). According to this observation, it may be argued that pSTAT3-activated tumours represent a more indolent tumour type. Activation of the STAT pathway has also been associated with EGFR mutations (Sordella *et al*, 2004), and has been proposed as a marker to identify patients to be treated with EGFR TKIs. The low prevalence of EGFR mutations in our patient cohort (4%) precluded the study of such an association in our study. Another interesting observation was the presence of a specific granular staining pattern for pERK, which was related with improved prognosis. Upon further analysis, this specific staining pattern was found to be correlated with the presence of KRAS mutations (see Supplementary information for details). The molecular basis for this correlation remains to be elucidated.

In summary, we showed that KRAS mutation, EGFR.C, E-cadherin.C, pSTAT3.N, pCMET.1003.C and pERK.gr were markers that were associated in a combined fashion with survival of NSCLC patients after taking stage into consideration. The good predictive value of EGFR mutations and poor predictive value of KRAS mutations with regard to EGFR TKI treatment was not reflected in terms of prognosis in our patient cohort. This study was conducted in an exploratory manner and the results may not be applicable to patients who receive adjuvant chemotherapy that has recently become standard for stage II and III disease. Confirmation of the impact of these markers in independent, adequately sized studies is necessary before considering these markers to be used for future evaluation of patient prognosis in clinical practice.

## Figures and Tables

**Figure 1:**
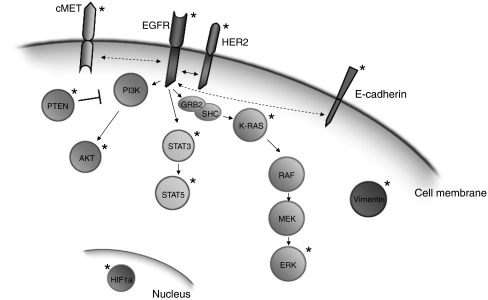
Overview of markers analysed in this study. A graphic display of a selection of EGFR pathway-related markers. The markers analysed in this study are indicated with an asterisk (^★^). Dashed lines indicate hypothesised interactions.

**Table 1:** Clinicopathological characteristics of the patients included in this study

	***n*=178**	
**Characteristics**	***n***	**(%)**
*Gender*		
Male	127	(71)
Female	51	(29)
		
*Histology*		
Adenocarcinoma	64	(36)
BAC	6	(3)
Squamous cell carcinoma	77	(43)
Large cell carcinoma	24	(14)
Others	7	(4)
		
*Smoking status*		
Never	3	(2)
Former	70	(39)
Current	60	(34)
Unknown	45	(25)
		
*Tumor stage*		
I	90	(51)
II	52	(29)
III	36	(20)
		
*Treatment*		
No treatment	133	(75)
Pre-operative chemotherapy	28	(16)
Post-operative chemotherapy	3	(2)
Post-operative radiotherapy	11	(6)
Pre-operative chemo/radiotherapy	3	(1)
		
*Type of surgery*		
Pneumonectomy	53	(30)
Lobectomy	111	(62)
Bilobectomy	8	(5)
Wedge resection	6	(3)
		
*Resection*		
Complete resection (R0)	123	(69)
Microscopic residue (R1)	36	(20)
Macroscopic residue (R2)	9	(5)
Uncertain	10	(6)

**Table 2:** Univariate association between individual parameters and survival

**Clinical and demographic markers**		**Genetic markers**		**Protein markers**				
					***P*-value per subcellular localisation**			
**Parameters**	***P*-value**	**Parameters**	***P*-value**	**Parameters**	**Membranous**	**Cytoplasmic**	**Granular**	**Nuclear**
Age	0.49							
R0 *vs* R1/R2	0.057	*EGFR FISH*		EGFR	0.63	0.10 (0.099^a^)	—	0
Adenocarcinoma *vs* all other	0.54	No gain *vs* other	0.15	HER2	1	0.33	—	—
BAC *vs* all other	0.43	High polysomy *vs* other	0.13	pAKT.473	—	0.22	—	0.62
Squamous cell *vs* all other	0.42	Amplification *vs* other	0.72	pAKT.309	0.53	1	—	0.95
Large cell carcinoma *vs* all other	0.9			PTEN	—	0.63	—	0.69
Smoking (non *vs* current *vs* Former)	0.79	*EGFR mutation*		pERK	—	0.36	0.011	0.39
Stage (I *vs* II *vs* III)	0.04	WT *vs* other	0.15	pSTAT3	0.97	0.50	—	0.056 (0.11^b^)
Stage (I *vs* II/III)	0.015 (0.03^c^)	SNP *vs* other	0.70	pSTAT5	0.76	0.31	—	0.33
Pre-operative CT *vs* not	0.45	Mutant *vs* other	0.33	E-cadherin	0.32	0.031	—	—
Post-operative CT *vs* not	0.5			Vimentin	0.29	0.80	—	0.45
Post-operative RT *vs* not	0.15	*KRAS mutation*		HIF-1*α*	—	—	—	0.55
Pre-operative chemo-radiation *vs* not	0.031^d^	WT *vs* other	0.08	pCMET.1003	0.8	0.11	—	—
Other diseases *vs* not	0.37	SNP *vs* other	0.84	pCMET.1349	—	0.49	—	0.69
		Mutant *vs* other	0.08	pCMET.1230	0.88	0.90	—	0.68
				pCMET.1365	0.55	0.87	—	0.86

Abbreviations: CT=chemotherapy; EGFR=epidermal growth factor receptor; FISH=fluorescent *in situ* hybridization; R0, R1, R2=resection1, 2, 3; RT=radiotherapy; SNP=single nucleotide polymorphism; WT=wild type.

a EGFR C: *P*=0.10 for 0–100 *vs* 160–300; *P*=0.20 after adjustment for new division in data.

b pSTAT3 N: *P*=0.056 for 0–200 *vs* 210+; *P*=0.11 after adjusting *P*-value.

c *P*=0.03 after adjustment for tests leading to re-grouping.

d Only three patients received pre-operative chemo-radiation; thus this parameter will not be considered in any other analyses.

**Table 3:** Prognostic significance from Cox model using backward selection

**Variable**	**Parameter estimate**	***P*-value**	**Hazard Ratio**	**95% CI for HR**
Stage (I *vs* II/III)	0.55	0.035	1.73	1.04–2.87
KRAS mutation	−0.95	0.016	0.39	0.18–0.84
EGFR C	0.9	0.015	2.45	1.19–5.02
E-cadherin.C	−0.96	0.0069	0.38	0.19–0.77
pSTAT3.N	−1.18	0.0006	0.31	0.16–0.60
pCMET.1003.C	0.78	0.0049	2.16	1.27–3.73

Abbreviations: EGFR=epidermal growth factor receptor; CI=confidence interval; HR=Hazard ratio.
