# Supplementary material for: Combined assessment of EGFR pathway-related molecular markers and prognosis of NSCLC patients
Source: Br J Cancer. 2008 Dec 2;100(1):145–52. doi: 10.1038/sj.bjc.6604781 (PMC2634682; doi:10.1038/sj.bjc.6604781)

**Supplementary Information**

**Association between granular pERK staining and KRAS mutations**

We observed an association between KRAS mutations and the presence of a typical granular pERK staining. In Figure S1 examples of a diffuse pERK staining in the nucleus and cytoplasm (panel A) and a granular pERK staining in the cytoplasm (panel B) can be observed. In panel C it can be observed that the proportion of patients having the granular staining pattern is higher in patients harboring a KRAS mutation than in patients having wild type KRAS status. The molecular basis for the correlation between KRAS mutation and pERK granular staining pattern should be investigated in future fundamental research.


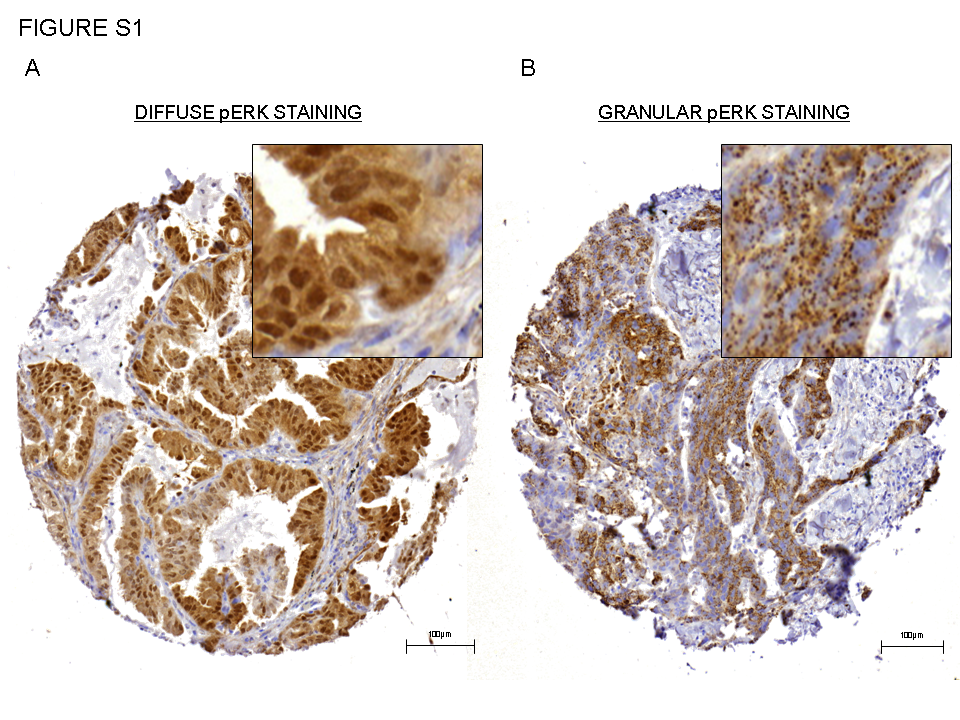

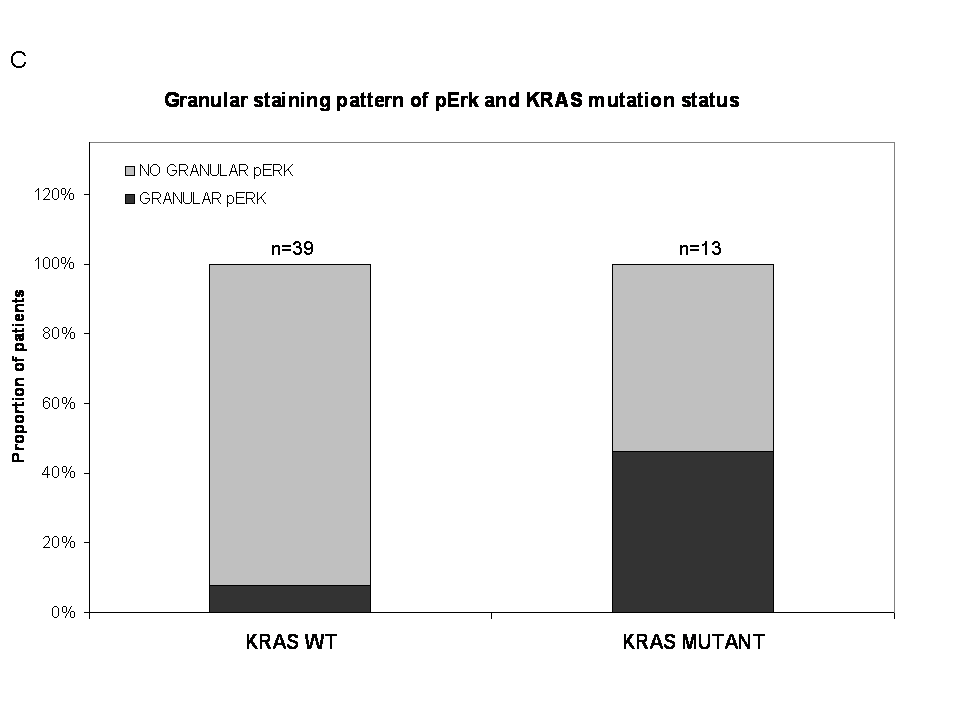

Supplement: Supplementary Information [file 6604781x1.doc]
